# Cross-sectional association between a CLHLS-based diet quality score and screening-defined elevated depressive symptoms among Chinese older adults

**DOI:** 10.3389/fnut.2026.1833110

**Published:** 2026-06-26

**Authors:** Zhaoquan Jiang, Zhaoxu Xu, Mingyue Zhou, Huijun Zhang, Shixue Zhou

**Affiliations:** 1 School of Nursing, Jinzhou Medical University, Jinzhou, Liaoning, China; 2 Editorial Department (Social Sciences Edition), Jinzhou Medical University, Jinzhou, Liaoning, China

**Keywords:** Chinese Longitudinal Healthy Longevity Study, cross-sectional study, diet quality score, older adults, screening-defined elevated depressive symptoms

## Abstract

**Background:**

Depressive symptoms are common in later life and may be related to dietary behaviours. However, dietary habits and depressive symptoms may influence each other, and cross-sectional data cannot establish temporal direction. This study examined the cross-sectional association between a Chinese Longitudinal Healthy Longevity Study (CLHLS)-based diet quality score and screening-defined elevated depressive symptoms among Chinese older adults.

**Methods:**

This cross-sectional study used data from the 2018 CLHLS. Dietary behaviour was assessed using self-reported intake frequency of 13 food groups, with items coded so that higher values indicated higher intake frequency. A CLHLS-based diet quality score was then constructed. Elevated depressive symptoms were assessed using the CESD-10. Multivariable logistic regression was used to examine the association between diet quality and elevated depressive symptoms. False discovery rate correction was applied in exploratory food-group analyses. Sensitivity analyses included multiple imputation with 20 datasets and a modified CESD-10 score excluding the sleep-quality item.

**Results:**

Among 15,874 participants in the 2018 CLHLS, 12,069 adults aged 60 years or older had complete dietary and depressive symptom data. In the fully adjusted complete-case model (*n* = 9,086), each five-point increase in the CLHLS-based diet quality score was associated with lower odds of elevated depressive symptoms (OR = 0.877, 95% CI: 0.844–0.912, *p* < 0.001). Although statistically significant, the association was modest in magnitude, and explanatory power was limited. The multiple-imputation analysis yielded a similar estimate (pooled OR = 0.890, 95% CI: 0.860–0.922, *p* < 0.001). The association also remained when depressive symptoms were analysed using the modified CESD-10 score. In exploratory food-group analyses, higher reported intake frequencies of fruit and vegetables were associated with lower odds of elevated depressive symptoms after adjustment.

**Conclusion:**

The CLHLS-based diet quality score was weakly associated with screening-defined elevated depressive symptoms. These findings should be interpreted as descriptive and hypothesis-generating, rather than causal or sufficient to support clinical or public health dietary recommendations. Longitudinal studies using validated dietary assessment tools are needed.

## Introduction

Population ageing has become a major public health and social challenge in China and worldwide (1). As people grow older, they are more likely to experience chronic diseases, functional decline, bereavement, reduced social participation, and social isolation, all of which may increase vulnerability to depressive symptoms (2). Depressive symptoms in later life are associated with poorer quality of life, disability, increased healthcare use, and greater care burden. Against this background, modifiable lifestyle factors, such as diet, have received increasing attention as potentially relevant correlates of mental health in older populations (3).

In nutritional psychiatry, research has gradually moved beyond single nutrients toward overall dietary patterns. This shift is important because foods are consumed in combination, and their associations with mental health may reflect the combined effects of multiple nutrients, bioactive compounds, eating habits, and broader lifestyle contexts (4, 5). Previous studies have suggested that healthier dietary patterns, such as Mediterranean-style diets, DASH-style diets, and higher diet-quality scores, are often associated with fewer depressive symptoms (6–9). A meta-analysis focused on older adults also reported that healthy dietary patterns were associated with a lower risk of depression, although substantial heterogeneity was observed across studies (10). Recent cohort evidence among older adults has similarly suggested that higher diet quality may be associated with lower depressive symptom burden (11). However, findings are not entirely consistent across populations, and evidence from Chinese older adults remains more limited than that from Western populations.

This study used dietary information available in the Chinese Longitudinal Healthy Longevity Study (CLHLS). The CLHLS dietary module records intake frequency for selected food groups, rather than detailed information on portion size, total energy intake, nutrient density, or cooking methods. Therefore, the diet quality score used in this study should be understood as a CLHLS-based summary of available food-frequency items rather than as a standard Healthy Eating Index instrument. Standard HEI instruments are designed to assess adherence to dietary guidelines using more detailed dietary intake data, whereas the CLHLS-based score reflects the frequency of consumption of 13 available food groups (12). This distinction is important when interpreting both the overall diet quality score and individual food-group associations.

Using data from the 2018 wave of the CLHLS, this study examined the cross-sectional association between a CLHLS-based diet quality score and screening-defined elevated depressive symptoms among Chinese older adults. It also evaluated associations for individual food groups and assessed the robustness of the findings after broader adjustment for sociodemographic, lifestyle, health-related, cognitive, functional, and social factors. Since dietary behaviour and depressive symptoms were measured at the same time point, the analysis focused on describing associations and their consistency across models rather than determining temporal or causal relationships.

## Methods

### Study design and participants

This study was a cross-sectional analysis of the 2018 wave of the Chinese Longitudinal Healthy Longevity Study (CLHLS), a large population-based survey of older adults in China. The CLHLS collects information on sociodemographic characteristics, lifestyle behaviours, dietary habits, physical health, cognitive function, mental health-related information, and family and social circumstances through standardised household interviews conducted by trained interviewers (13). When participants were unable to complete the interview independently, information was provided by a spouse or another close family member.

**Figure 1:**
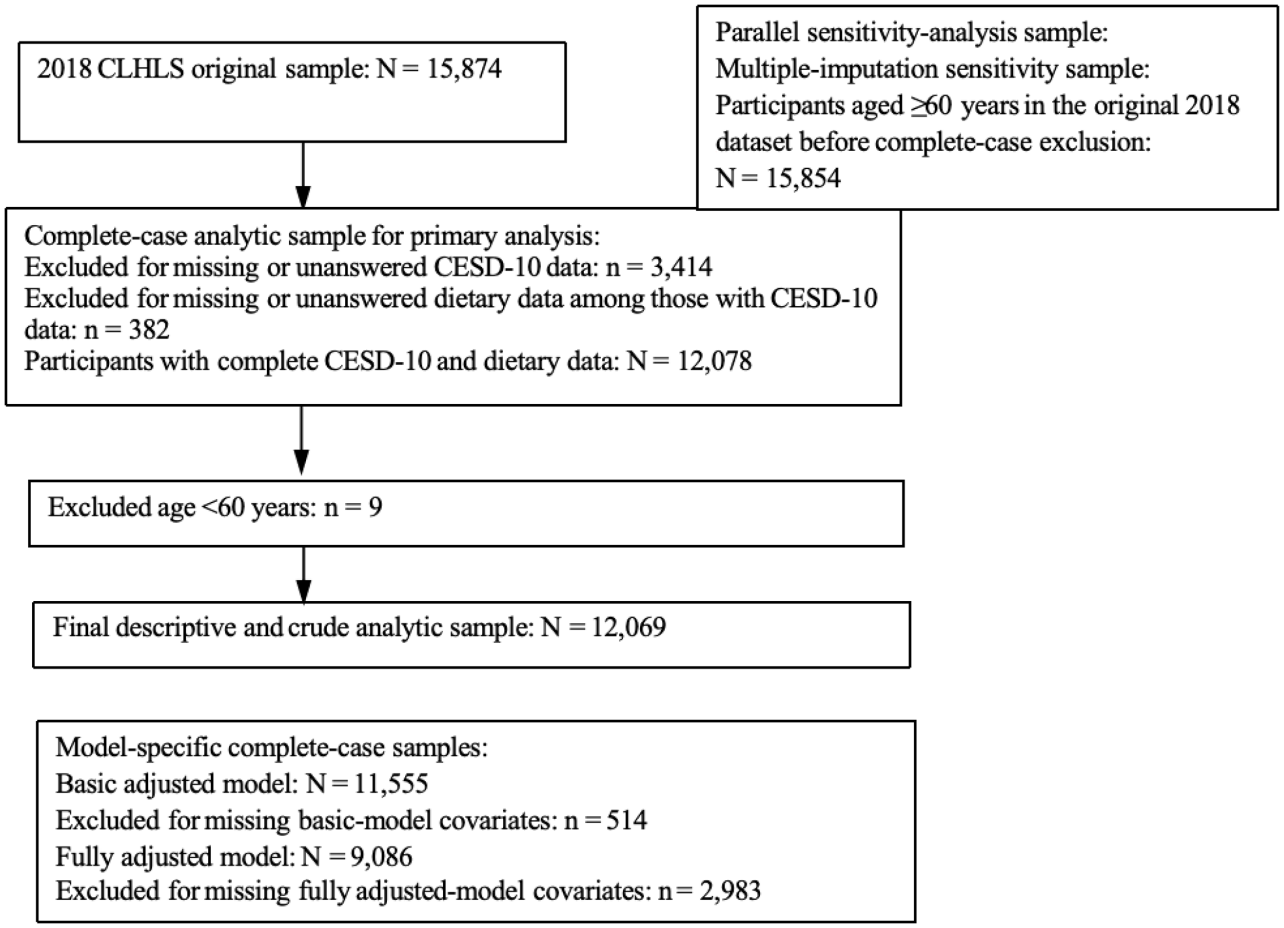
Flow diagram of participant selection and analytic samples.

The 2018 wave of the CLHLS included 15,874 participants. For the complete-case primary analysis, participants with missing or unanswered depressive-symptom data were first excluded (*n* = 3,414), followed by participants with missing or unanswered dietary data among those with depressive-symptom data (*n* = 382). This left 12,078 participants with complete key exposure and outcome variables. Since the present analysis focused on older adults, 9 participants younger than 60 years in this complete-key-variable sample were excluded, leaving 12,069 participants aged 60 years or older for descriptive and crude analyses. Regression models were conducted using complete cases for the variables included in each model. The basic adjusted model included 11,555 participants after excluding 514 participants with missing basic-model covariates. The fully adjusted model included 9,086 participants after excluding 2,983 participants with missing fully adjusted model covariates. In addition, a multiple-imputation sensitivity analysis was conducted among participants aged 60 years or older in the original 2018 dataset before complete-case exclusion (*N* = 15,854). The participant selection process and analytic samples are shown in Figure 1.

The CLHLS was approved by the Biomedical Ethics Committee of Peking University (IRB00001052-13074), and written informed consent was obtained from all participants or their legal representatives (13). This study used de-identified secondary data and did not involve direct contact with participants.

### Dietary behaviour and CLHLS-based diet quality score

Dietary behaviour was assessed using self-reported intake frequency of 13 food groups available in the CLHLS questionnaire: fruit, vegetables, meat, fish, eggs, beans, salt-preserved vegetables, sugar, tea, garlic, nuts, mushrooms or algae, and milk products. For fruit and vegetables, response options ranged from “rarely or never” to “almost every day.” For the remaining food groups, five ordered frequency categories were used, also ranging from “rarely or never” to “almost every day.” Food-frequency variables were recoded where necessary so that higher values consistently represented higher reported intake frequency.

A CLHLS-based diet quality score was constructed from these 13 food groups. Higher intake frequencies of generally recommended foods were assigned higher scores, whereas salt-preserved vegetables and sugar were reverse-scored because frequent consumption of these items is generally considered less favourable in diet-quality assessment (14, 15). The total score ranged from 0 to 50, with higher scores indicating more favourable diet quality within the limits of the CLHLS dietary questionnaire. The scoring criteria are shown in Table 1.

**Table 1:** Scoring criteria for the CLHLS-based diet quality score.

Food group	Scoring	Component range	Direction
Fruit	Rarely/never = 0; occasionally = 1; quite often = 2; almost every day = 3	0–3	Higher frequency scored higher
Vegetables	Rarely/never = 0; occasionally = 1; quite often = 2; almost every day = 3	0–3	Higher frequency scored higher
Meat	Rarely/never = 0 to almost every day = 4	0–4	Higher frequency scored higher
Fish	Rarely/never = 0 to almost every day = 4	0–4	Higher frequency scored higher
Eggs	Rarely/never = 0 to almost every day = 4	0–4	Higher frequency scored higher
Beans	Rarely/never = 0 to almost every day = 4	0–4	Higher frequency scored higher
Tea	Rarely/never = 0 to almost every day = 4	0–4	Higher frequency scored higher
Garlic	Rarely/never = 0 to almost every day = 4	0–4	Higher frequency scored higher
Nuts	Rarely/never = 0 to almost every day = 4	0–4	Higher frequency scored higher
Mushrooms or algae	Rarely/never = 0 to almost every day = 4	0–4	Higher frequency scored higher
Milk products	Rarely/never = 0 to almost every day = 4	0–4	Higher frequency scored higher
Salt-preserved vegetables	Almost every day = 0 to rarely/never = 4	0–4	Reverse-scored
Sugar	Almost every day = 0 to rarely/never = 4	0–4	Reverse-scored

The total score ranged from 0 to 50. Food-frequency variables were recoded where necessary so that higher values represented higher reported intake frequency before scoring. The score summarises available CLHLS food-frequency items and is not equivalent to standard Healthy Eating Index instruments based on portion size, nutrient density, or dietary guideline components.

This score was derived from brief food-frequency categories and did not include portion size, total energy intake, nutrient density, cooking methods, food subtype, freshness, or detailed dietary guideline components. Therefore, it was used as a CLHLS-based summary indicator of the available dietary items rather than as a standard Healthy Eating Index instrument or a comprehensive measure of nutrient adequacy. The score has not been validated against detailed dietary assessment methods such as repeated 24-h dietary recalls, food diaries, or comprehensive food-frequency questionnaires with portion size.

Food-specific associations were examined in two ways: first, in separate fully adjusted models for each food group, and then in a simultaneous model including all 13 food groups. As a sensitivity analysis, the diet quality score was recalculated after excluding salt-preserved vegetables and sugar.

### Depressive symptoms

Depressive symptoms were assessed using the 10-item Center for Epidemiologic Studies Depression Scale available in the CLHLS. Total scores range from 0 to 30, with higher scores indicating more severe depressive symptoms. Screening-defined elevated depressive symptoms were defined primarily as a CESD-10 score of 10 or higher. Additional analyses were conducted using CESD-10 cutoffs of ≥12 and ≥15 to examine whether the results varied across different screening thresholds. A modified continuous depressive-symptom score excluding the sleep-quality item was also examined because sleep-related symptoms may partly overlap with physical health burden in older adults. Throughout the manuscript, the outcome is described as screening-defined elevated depressive symptoms rather than clinically diagnosed depression (16–18). The internal consistency of the CESD-10 in the final analytic sample was Cronbach’s alpha = 0.822.

### Covariates

Covariates were selected according to their potential associations with both dietary behaviour and depressive symptoms in older adults. The basic model included age, gender, marital status, regular exercise, current smoking, and current alcohol drinking. The fully adjusted model additionally included residence, years of schooling, financial support sufficiency, self-rated health, activities of daily living limitation count, chronic disease count, cognitive function score, co-residence status, and current social activity participation.

Activities of daily living were summarised using six basic self-care items: bathing, dressing, toileting, indoor transferring, continence, and feeding. A limitation count was calculated as the number of activities requiring partial or substantial assistance. Chronic disease count was calculated from self-reported physician-diagnosed conditions available in the CLHLS. Cognitive function was summarised using available orientation, registration, calculation, recall, and language items, with higher scores indicating better cognitive performance. Interview respondent status was used in sensitivity analysis to exclude participants whose information was provided by proxy respondents.

### Statistical analysis

Descriptive statistics were used to summarise participant characteristics. Continuous variables are presented as means and standard deviations, and categorical variables are presented as frequencies and percentages. Characteristics of included and excluded participants were compared using *t*-tests or chi-squared tests, as appropriate, to describe potential differences related to missing data.

Logistic regression models were used to examine the association between the CLHLS-based diet quality score and screening-defined elevated depressive symptoms. Odds ratios (ORs), 95% confidence intervals (CIs), *p* values, and Nagelkerke *R*^2^ were reported. The diet quality score was scaled per five-point increase to improve interpretability. Model 1 was unadjusted. Model 2 was adjusted for demographic and lifestyle variables, including age, gender, marital status, regular exercise, current smoking, and current alcohol drinking. Model 3 was further adjusted for residence, education, financial support sufficiency, self-rated health, activities of daily living limitation count, chronic disease count, cognitive function score, co-residence status, and social activity participation. The primary regression analyses were conducted using complete cases for the variables included in each model.

Exploratory associations between individual food groups and elevated depressive symptoms were examined in separate fully adjusted models. The Benjamini–Hochberg false discovery rate method was used to account for multiple testing across the 13 food-group analyses. A simultaneous model including all 13 food groups was then fitted to examine whether food-specific associations remained when the other food groups were considered together.

Subgroup analyses were conducted by age group, categorised as 60–74, 75–89, and ≥90 years, and by residence, categorised as city, town, and rural. Interaction terms were included to assess whether the association between diet quality and elevated depressive symptoms differed across these groups.

Sensitivity analyses were performed by recalculating the diet quality score after excluding salt-preserved vegetables and sugar, excluding participants with limitations in three or more activities of daily living, treating the CESD-10 score as a continuous outcome, using a modified continuous depressive-symptom score excluding the sleep-quality item, modelling quartiles of the CLHLS-based diet quality score, applying alternative CESD-10 thresholds of ≥12 and ≥15, excluding participants with poor or very poor self-rated health, and excluding participants with any chronic disease. Additional restricted analyses were conducted among participants with no ADL limitations, participants without proxy respondents, and participants meeting both conditions. Subgroup, sensitivity, and restricted analyses were exploratory and were reported without additional adjustment for multiple comparisons.

### Multiple imputation sensitivity analysis

To assess the influence of missing covariate data, multiple imputation was performed as a sensitivity analysis among participants aged 60 years or older in the original 2018 dataset before complete-case exclusion (*N* = 15,854) using fully conditional specification with 20 imputed datasets. The imputation model included the CLHLS-based diet quality score, screening-defined elevated depressive symptoms, continuous CESD-10 score, and all covariates used in the fully adjusted model: age, gender, marital status, regular exercise, current smoking, current alcohol drinking, residence, years of schooling, financial support sufficiency, self-rated health, activities of daily living limitation count, chronic disease count, cognitive function score, co-residence status, and current social activity participation. Proxy respondent status was included as an auxiliary variable because it was related to data quality and missingness.

Continuous variables were imputed using predictive mean matching, binary variables using logistic regression, and categorical variables using logistic or multinomial models, as appropriate. Imputed values for the CLHLS-based diet quality score were checked to ensure that they remained within the possible score range of 0–50. Regression models were fitted separately in each imputed dataset, and pooled estimates were calculated using Rubin’s rules. The complete-case analysis was retained as the primary analysis, and the multiple-imputation analysis was used to assess the robustness of the findings. Multiple imputation was conducted using the mice package in R.

## Results

### Participant characteristics and missing data

The final descriptive analytic sample included 12,069 participants aged 60 years or older. The mean age was 83.25 years (SD = 11.10), and 53.4% were women. Overall, 7,853 participants (65.1%) met the screening definition for elevated depressive symptoms. The mean CLHLS-based diet quality score was 26.38 (SD = 7.08), and the mean CESD-10 score was 11.94 (SD = 5.86). Participant characteristics are shown in Table 2.

**Table 2:** Participant characteristics (*N* = 12,069).

Characteristic	Category	Value
Age, years	Mean (SD)	83.25 (11.10)
CLHLS-based diet quality score	Mean (SD)	26.38 (7.08)
CESD-10 score	Mean (SD)	11.94 (5.86)
Elevated depressive symptoms	CESD-10 ≥ 10	7,853 (65.1)
Gender	Female	6,450 (53.4)
Residence	City	2,839 (23.5)
Residence	Town	3,959 (32.8)
Residence	Rural	5,271 (43.7)
Currently married	Yes	5,376 (44.5)
Regular exercise	Yes	4,123 (34.2)
Current smoking	Yes	1,941 (16.1)
Current drinking	Yes	1,845 (15.3)
Financial support sufficient	Yes	10,421 (86.3)
Social activity participation	Yes	1,836 (15.2)
Any ADL limitation	Yes	2,101 (17.4)
Proxy respondent	Yes	3,059 (25.3)
Education, years	Mean (SD)	3.72 (4.45)
Chronic disease count	Mean (SD)	1.61 (1.70)
Cognitive function score	Mean (SD)	28.90 (6.38)

Table 3 compares the included and excluded participants on the available variables. Compared with the included participants, excluded participants were older, more likely to be female, had poorer self-rated health, and had a higher prevalence of ADL limitation. These differences suggested potential non-random missingness and supported the use of multiple imputation as a sensitivity analysis.

**Table 3:** Comparison of included and excluded participants on available variables.

Variable	Included	Excluded	*p* value
Age	83.25	92.47	<0.001
Female	53.4%	65.7%	<0.001
Self-rated health	2.54	2.75	<0.001
Any ADL limitation	18.1%	53.6%	<0.001
Any chronic disease	72.8%	68.4%	<0.001
Diet quality score	26.38	23.48	<0.001
CESD-10 score	11.94	12.29	0.229

Values for excluded participants were calculated using available non-missing data for each variable; denominators may therefore vary across variables.

### Association between diet quality and elevated depressive symptoms

The CLHLS-based diet quality score was negatively correlated with continuous CESD-10 score (*r* = −0.229). Table 4 presents sequential logistic regression models. In the crude model, each five-point increase in diet quality score was associated with lower odds of elevated depressive symptoms (OR = 0.758, 95% CI: 0.738–0.779, *p* < 0.001; Nagelkerke *R*^2^ = 0.045). In the fully adjusted model, the corresponding OR was 0.877 (95% CI: 0.844–0.912, *p* < 0.001; Nagelkerke *R*^2^ = 0.188).

**Table 4:** Sequential logistic regression models for diet quality and elevated depressive symptoms.

Model	*N*	OR per 5-point increase	95% CI	*p* value	Nagelkerke *R*^2^
Crude	12,069	0.758	0.738–0.779	<0.001	0.045
Basic adjusted	11,555	0.810	0.786–0.834	<0.001	0.077
Fully adjusted	9,086	0.877	0.844–0.912	<0.001	0.188

The outcome was screening-defined elevated depressive symptoms. The basic adjusted model included age, gender, marital status, regular exercise, current smoking, and current alcohol drinking. The fully adjusted model additionally included residence, education, financial support sufficiency, self-rated health, ADL limitation count, chronic disease count, cognitive function score, co-residence status, and social activity participation.

### Food-group associations

Exploratory food-group associations are shown in Table 5. Higher reported intake frequencies of fruits and vegetables were inversely associated with elevated depressive symptoms. In separate fully adjusted models with FDR correction, higher intake frequencies of fruit, vegetables, eggs, tea, garlic, nuts, mushrooms or algae, and milk products were associated with lower odds of elevated depressive symptoms; fish showed a positive association.

**Table 5:** Separate fully adjusted models for individual food groups.

Food group	*N*	OR per category increase	95% CI	*p* value	FDR-adjusted *p*
Fruit	9,086	0.891	0.851–0.933	<0.001	<0.001
Vegetables	9,086	0.747	0.696–0.801	<0.001	<0.001
Meat	9,086	1.007	0.967–1.048	0.746	0.753
Fish	9,086	1.069	1.029–1.110	<0.001	0.001
Eggs	9,086	0.930	0.894–0.968	<0.001	0.001
Beans	9,086	1.019	0.981–1.060	0.330	0.390
Salt-preserved vegetables	9,086	1.022	0.991–1.055	0.171	0.222
Sugar	9,086	1.005	0.973–1.038	0.753	0.753
Tea	9,086	0.956	0.929–0.985	0.003	0.005
Garlic	9,086	0.946	0.916–0.978	<0.001	0.001
Nuts	9,086	0.921	0.888–0.955	<0.001	<0.001
Mushrooms or algae	9,086	0.866	0.831–0.902	<0.001	<0.001
Milk products	9,086	0.945	0.917–0.974	<0.001	0.001

Each row comes from a separate fully adjusted logistic regression model. ORs reflect one-category increases in reported intake frequency. FDR, false discovery rate.

The simultaneous food-group model is shown in Table 6. A simultaneous fully adjusted model including all 13 food groups was also fitted to examine whether food-specific associations remained when the other food groups were considered together. In this model, higher intake frequencies of fruit, vegetables, eggs, tea, and mushrooms or algae remained associated with lower odds of elevated depressive symptoms. Fish and beans showed positive associations, whereas meat, salt-preserved vegetables, sugar, garlic, nuts, and milk products were not statistically significant. The simultaneous model had a Nagelkerke *R*^2^ of 0.206.

**Table 6:** Simultaneous fully adjusted model including all 13 food groups.

Food group	OR per category increase	95% CI	*p* value
Fruit	0.935	0.891–0.983	0.008
Vegetables	0.766	0.712–0.824	<0.001
Meat	1.022	0.978–1.069	0.326
Fish	1.117	1.071–1.165	<0.001
Eggs	0.937	0.897–0.978	0.003
Beans	1.066	1.021–1.112	0.004
Salt-preserved vegetables	1.033	1.000–1.068	0.052
Sugar	1.018	0.984–1.053	0.296
Tea	0.967	0.938–0.997	0.029
Garlic	0.970	0.937–1.004	0.079
Nuts	0.967	0.928–1.007	0.108
Mushrooms or algae	0.887	0.847–0.930	<0.001
Milk products	0.973	0.942–1.006	0.107

All 13 food groups were entered simultaneously in one fully adjusted logistic regression model. The model included the same covariates as the fully adjusted model in Table 4. *N* = 9,086; Nagelkerke *R*^2^ = 0.206.

### Subgroup analyses

Subgroup analyses by age group and residence are shown in Table 7. The inverse association between the CLHLS-based diet quality score and elevated depressive symptoms was observed across all age groups and residence groups. There was no evidence of statistically significant interaction by age group (*p* for interaction = 0.881) or residence (*p* for interaction = 0.109). These subgroup analyses were descriptive and were not adjusted for multiple comparisons.

**Table 7:** Subgroup analyses of the association between diet quality and elevated depressive symptoms.

Subgroup variable	Subgroup	*N*	OR per 5-point increase	95% CI	*p* value	*p* for interaction
Age group	60–74	2,486	0.837	0.777–0.901	<0.001	0.881
Age group	75–89	3,648	0.877	0.825–0.932	<0.001	0.881
Age group	≥90	2,952	0.916	0.853–0.982	0.014	0.881
Residence	City	2,366	0.889	0.825–0.959	0.002	0.109
Residence	Town	2,962	0.896	0.835–0.962	0.002	0.109
Residence	Rural	3,758	0.854	0.804–0.907	<0.001	0.109

All subgroup models used the fully adjusted covariate set. Age was omitted from age-stratified models, and residence was omitted from residence-stratified models. Results were not adjusted for multiple comparisons.

### Sensitivity analyses

Sensitivity analyses supported the direction of the primary association (Table 8). In the multiple-imputation analysis using 20 imputed datasets based on the original 2018 sample aged 60 years or older, the pooled OR was 0.890 (95% CI: 0.860–0.922, *p* < 0.001). The association also remained in analyses using stricter CESD-10 thresholds, and when depressive symptoms were analysed as a continuous CESD-10 score. When the sleep-quality item was excluded from the CESD-10 score, higher diet quality remained associated with a lower modified depressive-symptom score (*β* = −0.341, 95% CI: −0.424 to −0.259, *p* < 0.001). Results were also consistent in analyses restricted by self-rated health, chronic disease status, ADL limitation, and proxy response.

**Table 8:** Sensitivity analyses.

Analysis	*N*	Estimate type	Estimate	95% CI	*p* value
Main fully adjusted complete-case analysis	9,086	OR	0.877	0.844–0.912	<0.001
Multiple imputation, 20 datasets	15,854	OR	0.890	0.860–0.922	<0.001
Alternative score excluding salt-preserved vegetables and sugar	9,086	OR	0.891	0.858–0.925	<0.001
Excluding participants with ≥3 ADL limitations	8,394	OR	0.877	0.842–0.913	<0.001
Continuous CESD-10 score	9,086	β	−0.397	−0.484 to −0.310	<0.001
Modified CESD-10 score excluding sleep-quality item	9,086	*β*	−0.341	−0.424 to −0.259	<0.001
CESD-10 cutoff ≥12	9,086	OR	0.879	0.847–0.913	<0.001
CESD-10 cutoff ≥15	9,086	OR	0.891	0.855–0.929	<0.001
Excluding poor or very poor self-rated health	7,861	OR	0.879	0.845–0.916	<0.001
Excluding any chronic disease	2,362	OR	0.856	0.794–0.923	<0.001

The multiple-imputation analysis was conducted among participants aged 60 years or older in the original 2018 dataset before complete-case exclusion. Results from sensitivity analyses were not adjusted for multiple comparisons.

Additional restricted analyses were conducted among participants without ADL limitation, participants without proxy respondents, and participants meeting both criteria (Table 9). Higher intake frequencies of fruit and vegetables remained inversely associated with elevated depressive symptoms in these restricted analyses.

**Table 9:** Restricted analyses for fruit and vegetable findings.

Restricted sample	Food group	*N*	OR per category increase	95% CI	*p* value
No ADL limitation	Fruit	7,393	0.897	0.852–0.944	<0.001
No ADL limitation	Vegetables	7,393	0.731	0.674–0.792	<0.001
No proxy respondent	Fruit	6,675	0.880	0.834–0.929	<0.001
No proxy respondent	Vegetables	6,675	0.698	0.639–0.763	<0.001
No ADL limitation and no proxy respondent	Fruit	6,008	0.888	0.839–0.939	<0.001
No ADL limitation and no proxy respondent	Vegetables	6,008	0.695	0.632–0.763	<0.001

Each row comes from a separate fully adjusted logistic regression model. These restricted analyses were conducted to assess whether the fruit and vegetable findings were consistent after excluding participants with ADL limitations or proxy responses. Results were not adjusted for multiple comparisons.

## Discussion

This cross-sectional study examined the association between a CLHLS-based diet quality score and screening-defined elevated depressive symptoms among Chinese older adults. Higher diet quality was associated with lower odds of elevated depressive symptoms after adjustment for demographic, socioeconomic, lifestyle, health-related, cognitive, functional, living arrangement, and social activity variables. In the fully adjusted complete-case model, each five-point increase in the diet quality score was associated with an OR of 0.877 for elevated depressive symptoms. Although this association was statistically significant, its magnitude was modest, and the crude model explained only a limited proportion of variation in elevated depressive symptoms. The association was similar in the multiple-imputation analysis and remained in sensitivity analyses using stricter CESD-10 thresholds. It also remained when depressive symptoms were analysed using a modified CESD-10 score excluding the sleep-quality item, suggesting that the result was not explained solely by the sleep-related component of the depressive-symptom measure. Nevertheless, the cross-sectional design, limited explanatory power, and brief dietary assessment limit interpretation of temporal direction, clinical relevance, and public health significance.

Since dietary behaviour and depressive symptoms were measured at the same time point, the temporal sequence of the association cannot be established. Better dietary habits may be linked to a more favourable mental health status, but depressive symptoms may also influence eating behaviour through appetite changes, altered food preferences, reduced motivation for food preparation, and less regular eating patterns. Previous evidence has also suggested that reverse causality may partly explain observed associations between diet and depression (19). Health status may further complicate this relationship. Older adults with chronic disease, functional limitations, poorer self-rated health, chewing difficulties, appetite changes, or reduced capacity to prepare food may adjust their diets in response to illness or care needs. The present findings should therefore be interpreted as cross-sectional associations within a broader context of diet, health status, and depressive symptoms rather than as evidence of a simple directional relationship.

A notable feature of this study was the high proportion of participants classified as having elevated depressive symptoms. Overall, 65.1% met the CESD-10 ≥ 10 screening definition. This figure should be interpreted in relation to the age and health profile of the CLHLS sample and the measurement approach used in the survey. The CLHLS includes many very old adults, among whom frailty, functional limitations, bereavement, care dependence, and social isolation are common. In addition, depressive-symptom scales in older populations may partly overlap with physical health burden when items capture sleep, fatigue, appetite, or other somatic experiences. In the present analysis, the inverse association was still observed after excluding the sleep-quality item from the CESD-10 score, although an overlap between depressive symptoms and physical health burden cannot be ruled out completely. For this reason, the outcome is best described as screening-defined elevated depressive symptoms rather than clinically diagnosed depression.

The inverse association between overall diet quality and elevated depressive symptoms is generally consistent with previous observational research. A systematic review and meta-analysis reported that greater adherence to healthy dietary indices was associated with a lower risk of depressive outcomes (4). Prospective evidence has also suggested that higher diet quality may be associated with a lower risk of depression, although the magnitude and consistency of this association vary across studies (20). Evidence from Chinese older adults similarly supports a possible link between healthier dietary patterns and fewer depressive symptoms (21, 22), and comparable findings have been reported in non-Chinese older populations (23). These studies provide useful context for the present analysis, but they also underline that diet–depression associations may differ according to dietary assessment method, outcome definition, age structure, health status, and study design.

The CLHLS-based diet quality score should be interpreted in relation to the dietary information available in the survey. Standard Healthy Eating Index instruments are designed to assess adherence to dietary guidance using multiple dietary components and detailed intake information (10). In contrast, the score used in this study was constructed from 13 food-frequency items in the CLHLS and did not include portion size, total energy intake, nutrient density, cooking methods, food subtype, freshness, or other components commonly used in guideline-based dietary indices. Comprehensive food-frequency questionnaires usually include a broader food list and may collect usual portion size, whereas the CLHLS dietary module provides only brief frequency information for selected food groups (24). Therefore, the present score is best regarded as a summary of available CLHLS dietary items rather than as a validated Healthy Eating Index or a comprehensive measure of nutritional adequacy. Future use of this score would be strengthened by validation against more detailed dietary assessment methods, such as repeated 24-h dietary recalls, food diaries, or food-frequency questionnaires that include portion size and food subtypes.

The food-group analyses provided additional context for interpreting the overall score. Higher reported intake frequencies of fruit and vegetables were inversely associated with elevated depressive symptoms, which is broadly consistent with much of the existing literature, although findings vary across populations, dietary assessment methods, and study designs (25–29). Other food groups showed more heterogeneous associations. In separate fully adjusted models, higher intake frequencies of eggs, tea, garlic, nuts, mushrooms or algae, and milk products were associated with lower odds of elevated depressive symptoms, whereas fish showed a positive association. In the simultaneous food-group model, fruits, vegetables, eggs, tea, and mushrooms or algae remained inversely associated with elevated depressive symptoms, while fish and beans showed positive associations. These differences suggest that individual food-group findings should be interpreted within the broader social, cultural, and health context of eating in later life, including regional dietary culture, income, food accessibility, family support, oral health, appetite, disease management, and cooking practices.

The food-specific findings should be interpreted as exploratory descriptive associations. Although several food groups showed statistically significant associations, these estimates may be influenced by residual confounding, correlated dietary behaviours, reverse causation, and exposure misclassification. The positive association observed for fish intake requires particular caution because fish consumption is often considered favourable in dietary guidance and has frequently been discussed as potentially beneficial in nutritional psychiatry. In the present analysis, this finding may also reflect regional dietary practices, food preparation methods, disease-related dietary changes, or limitations of the brief CLHLS dietary module. The CLHLS questionnaire records intake frequency but not the amount consumed, cooking method, freshness, food subtype, or accompanying ingredients. Similarly, the vegetable item does not distinguish fresh vegetables from pickled, salted, or heavily cooked vegetables. These limitations mean that the food-group results should not be interpreted as evidence that any specific food directly increases or decreases depressive symptoms, nor do they provide a basis for food-specific dietary recommendations or biological interpretation.

Salt-preserved vegetables and sugar were reverse-scored in the diet quality score because frequent consumption of these items is generally considered less favourable from a diet-quality perspective. In the food-group analysis, salt-preserved vegetables were not significantly associated with elevated depressive symptoms after adjustment and FDR correction. The association between the overall diet quality score and elevated depressive symptoms remained when salt-preserved vegetables and sugar were excluded from the score. This suggests that the main association was not driven by these two reverse-scored components, although the interpretation of the score remains limited by the brief dietary assessment.

Subgroup analyses showed that the inverse association between diet quality and elevated depressive symptoms was observed across all age groups, including participants aged 90 years or older. There was no statistically significant interaction by age group. The estimates were also inverse across city, town, and rural residence groups, with no statistically significant interaction by residence. These findings suggest that the association did not differ materially by age group or residence. However, subgroup findings should be viewed as descriptive because they were not adjusted for multiple comparisons and require confirmation in longitudinal studies.

The comparison between included and excluded participants suggested potential selection bias. Compared with included participants, excluded participants were older, more likely to be female, had poorer self-rated health, and had a higher prevalence of ADL limitation. These differences suggest that missingness was unlikely to be completely random. To assess the influence of missing covariate data, multiple imputation with 20 imputed datasets was performed as a sensitivity analysis. The pooled estimate was similar to the complete-case result, supporting the direction of the primary association. Nevertheless, multiple imputation cannot fully remove the possibility of selection bias related to non-response or unmeasured factors.

This study has several strengths. It used a large national sample of Chinese older adults and examined both overall diet quality and individual food groups. The analyses included broad adjustment for demographic, socioeconomic, lifestyle, functional, cognitive, health-related, living arrangement, and social covariates. False discovery rate correction was applied to the separate food-group models. The study also compared included and excluded participants, used multiple imputation to assess the influence of missing covariate data, and conducted several sensitivity and restricted analyses, including analyses using stricter CESD-10 thresholds and analyses restricted by ADL limitation and proxy response.

Several limitations should be noted. First, the cross-sectional design limits conclusions about temporal sequence. Depressive symptoms may influence dietary behaviour, while unmeasured factors may also affect both diet and depressive symptoms. Second, dietary behaviour was assessed using brief self-reported intake frequency, without information on portion size, nutrient intake, total energy intake, cooking methods, food subtype, or freshness. Third, the most important exposure-related limitation is that the CLHLS-based diet quality score was derived from brief intake-frequency items rather than from a validated dietary assessment instrument. It did not capture portion size, total energy intake, nutrient density, food quality or freshness, cooking methods, or food subtypes such as fresh versus processed foods. The score should therefore be interpreted as a pragmatic summary of available CLHLS dietary items rather than as a validated nutritional exposure measure. This limitation substantially restricts clinical, nutritional, and mechanistic interpretation. Fourth, residual confounding remains possible despite broad adjustment, particularly for medication use, oral health, appetite, sleep, family support quality, food accessibility, and regional food culture. Fifth, some responses may have been provided by proxy respondents, which may introduce information bias for subjective items, although restricted analyses excluding proxy respondents showed a similar direction of association for fruits and vegetable intake. Sixth, included and excluded participants differed on several available characteristics, suggesting possible selection bias. Multiple imputation produced results consistent with complete-case analysis, but it cannot fully address bias from unmeasured missingness mechanisms. Finally, the proportion classified as having elevated depressive symptoms was high and may reflect the screening-based definition, the age and health profile of the analytic sample, and the survey measurement context. The outcome, therefore, represents screening-defined elevated depressive symptoms rather than clinically diagnosed depression.

Future studies may benefit from incorporating broader psychosocial and biological information. Psychological distress in older adults has been associated with the wellbeing, health status, social support, and physical functioning (30), and emerging research has considered possible links among diet, gut microbiota, and depression (31). These factors were not assessed in the present analysis and should not be inferred from the current findings.

Overall, the CLHLS-based diet quality score was inversely associated with screening-defined elevated depressive symptoms among Chinese older adults. The association remained after broad covariate adjustment, multiple imputation, alternative outcome definitions, and several restricted analyses. Food-specific findings were generally compatible with an inverse association for fruit and vegetable intake, but some heterogeneous food-group results, including the positive association observed for fish, require cautious interpretation. Given the cross-sectional design and the limited dietary information available in the CLHLS, these results are best viewed as descriptive and hypothesis-generating. Further validation of the diet score and longitudinal research is needed before stronger clinical or etiologic interpretations can be made.

## Conclusion

The CLHLS-based diet quality score was inversely associated with screening-defined elevated depressive symptoms among Chinese older adults in this cross-sectional analysis. The association remained in complete-case and multiple-imputation analyses, across alternative depressive-symptom definitions, and after excluding the sleep-quality item from the CESD-10 score. Higher reported intake frequencies of fruit and vegetables were also associated with lower odds of elevated depressive symptoms. These findings should be interpreted as descriptive and hypothesis-generating and are not sufficient to support clinical or public health dietary recommendations because the dietary score has not been validated against detailed dietary assessment methods, and the cross-sectional design cannot establish temporal direction. Longitudinal studies using validated dietary assessment tools and repeated mental health measurements are needed to clarify the relationship between diet quality and depressive symptoms in later life.

## Data Availability

The datasets presented in this study can be found in online repositories. The names of the repository/repositories and accession number(s) can be found below: doi: 10.18170/DVN/WBO7LK.
